# A pioneer calf foetus microbiome

**DOI:** 10.1038/s41598-020-74677-7

**Published:** 2020-10-19

**Authors:** Cesar E. Guzman, Jennifer L. Wood, Eleonora Egidi, Alison C. White-Monsant, Lucie Semenec, Sylvia V. H. Grommen, Elisa L. Hill-Yardin, Bert De Groef, Ashley E. Franks

**Affiliations:** 1 Department of Physiology, Anatomy and Microbiology, La Trobe University, Melbourne, VIC 3086 Australia; 2 Centre for Future Landscapes, La Trobe University, Melbourne, VIC 3086 Australia; 3 Department of Animal, Plant and Soil Sciences, Centre for Agribiosciences, La Trobe University, Melbourne, VIC 3086 Australia; 4 School of Health and Biomedical Sciences, RMIT University, Clements Drive, Bundoora, VIC 3083 Australia; 5 Hawkesbury Institute for the Environment, Western Sydney University, Richmond, NSW 2753 Australia; 6 Faculty of Science, Engineering and Technology, Swinburne University of Technology, Hawthorn, VIC 3122 Australia

**Keywords:** Developmental biology, Biodiversity, Microbial ecology, Gastrointestinal system, Applied microbiology, Microbial communities, Environmental microbiology

## Abstract

Foetus sterility until parturition is under debate due to reports of microorganisms in the foetal environment and meconium. Sufficient controls to overcome sample contamination and provide direct evidence of microorganism viability in the pre-rectal gastrointestinal tract (GIT) have been lacking. We conducted molecular and culture-based analyses to investigate the presence of a microbiome in the foetal GIT of calves at 5, 6 and 7 months gestation, while controlling for contamination. The 5 components of the GIT (ruminal fluid, ruminal tissue, caecal fluid, caecal tissue and meconium) and amniotic fluid were found to contain a pioneer microbiome of distinct bacterial and archaeal communities. Bacterial and archaeal richness varied between GIT components. The dominant bacterial phyla in amniotic fluid differed to those in ruminal and caecal fluids and meconium. The lowest bacterial and archaeal abundances were associated with ruminal tissues. Viable bacteria unique to the ruminal fluids, which were not found in the controls from 5, 6 and 7 months gestation, were cultured, subcultured, sequenced and identified. We report that the foetal GIT is not sterile but is spatially colonised before birth by a pioneer microbiome.

## Introduction

Colonisation of the gastrointestinal tract (GIT) by commensal microorganisms is critical for normal neonate health, development and intestinal mucosal immunity^1,2^. The neonatal GIT is thought to be colonised by vertical transmission of maternal microbiota^3^. Major factors that contribute to colonisation of the neonatal GIT are mode of delivery (vaginal or caesarean section)^4–6^, gestational age at birth (normal or preterm)^7,8^, source of milk^9,10^, and exposure to the surrounding environment^11^. Although the “sterile-foetus dogma” has persisted for many decades^12,13^, evidence suggests that microbial colonisation occurs in utero*.*

Foetal colonisation is supported by reports of bacterial species in the human placenta^14,15^, amniotic fluid^16,17^ and umbilical cord^18^, and distinct microbial populations in the meconium immediately after birth^19–21^. Particularly, neonate meconium has been considered indicative of bacterial colonisation of the foetal GIT. The meconium of full-term neonates comprised Gram-positive and Gram-negative bacteria, with *Enterococcus* and *Staphylococcus* species being the most common isolates^20^, whereas the meconium of 23–41-week-old infants comprised Proteobacteria and Firmicutes^22^.

There is currently no direct evidence that components of the foetal GIT (other than meconium) support a microbiome, nor when it might be colonised^22^. In addition, findings of microorganisms in the foetal environment have been criticised due to their reliance on molecular approaches, their use of samples with low microbial biomass that are prone to DNA contamination from kits, a lack of appropriate contamination controls, and a lack of proof of bacterial viability^12^.

Calves have served as mammalian models to investigate microbiomes, host-bacterial interactions and gene expression, and innate and acquired immune responses^23,24^. In addition, foetal calves provide an advantage when investigating microbial colonisation of the foetal GIT due to their large size and ease of separation of GIT components. However, in all animal models used to study foetal colonization of the human microbiome, there are reported biological similarities and differences inherent to each species. The bovine and human gestation length is approximately nine months, but the placentas differ in their cell layers which are classified as *Epitheliochorial* and *Hemochorial*, respectively^25^. Despite these differences, genetic expression remains quite similar between placentas in bovines and humans^26^. The GIT of the bovine foetus differs from the human foetus by having three extra GIT compartments (rumen, reticulum and omasum) which occur before the abomasum (or stomach). Each of these compartments have fluid and tissue fractions^27^. At the moment of birth, the rumen of a calf has been reported to be sterile and is described as a rudimentary compartment because it is not yet anatomically or physiologically developed. There is minimal development of the papillae on the internal surface of the rumen, and the vagus nerve (responsible for the rumen contractions) and the process of rumination do not function until 2 weeks of age^28^.

In this study, we examined calf foetuses at 5, 6 and 7 months gestation to determine the presence of a microbiome within different components of the GIT (ruminal fluid, ruminal tissue, caecal fluid, caecal tissue and meconium) and amniotic fluid. We present direct evidence that (1) distinct bacterial and archaeal microbial communities are present in association with the tissues and in fluids of the rumen and caecum, meconium and amniotic fluid of calf foetuses at 5, 6 and 7 months gestation; (2) potential contaminants can be excluded as a source of these microorganisms; and (3) viable bacterial colonies can be cultured from the foetal ruminal fluid.

## Results

An investigation of the microbial contribution from kits, environment and sampling procedure was conducted to evaluate all potential contamination sources. After accounting for potential microbial contaminants, the presence of microorganisms was characterised in samples of 5 GIT components (ruminal fluid, ruminal tissue, caecal fluid, caecal tissue and meconium) and amniotic fluid from Angus × Friesian calf foetuses at 5, 6 and 7 months gestation (*n* = 4 per age).

### Controls for sample contamination

Controls included media sterility tests as well as sequencing controls of the columns and tubes used for genomic DNA extraction. Sample swabs were taken from the dissection table, amniotic sac, skin, as well as the mesenteric surfaces of rumen, caecum and rectum. Open air samples were taken by exposing sterile growth medium to the environment and extraction column controls were included in the metagenomic sequencing analysis to control for kit contamination (Table 1).

**Table 1:** Description of control samples and test results per foetus (*n* = 12).

Control sample description	Test result			
	Concentration of extracted DNA and qPCR	Anaerobic culture^a^	Aerobic culture^a^	Illumina MiSeq sequencing
	3 swabs	3 swabs	3 swabs	
Surface of dissection table swabbed after washing with 70% ethanol (*n* = 9)	< 0.5 ng/µL DNA qPCR: no amplification product detected	0 CFU	0 CFU	Not performed
External surface of amniotic sac swabbed after rinsing thrice with sterile PBS prior to opening (*n* = 9)	< 0.5 ng/µL DNA qPCR: no amplification product detected	0 CFU	0 CFU	Not performed
Skin of the foetus swabbed after rinsing thrice with sterile PBS prior to opening (*n* = 9)	< 0.5 ng/µL DNA qPCR: no amplification product detected	0 CFU	0 CFU	Not performed
External (mesenteric) surfaces of the caecum swabbed prior to opening (*n* = 9)	< 0.5 ng/µL DNA qPCR: no amplification product detected	0 CFU	0 CFU	Not performed
External (mesenteric) surfaces of the rumen swabbed prior to opening (*n* = 9)	< 0.5 ng/µL DNA qPCR: no amplification product detected	0 CFU	0 CFU	Not performed
External (mesenteric) surfaces of the rectum swabbed prior to opening (*n* = 9)	< 0.5 ng/µL DNA qPCR: no amplification product detected	0 CFU	0 CFU	Not performed
Sampling tubes containing anaerobic medium opened to environment during foetal sampling. Samples incubated at 37 °C and CFUs reported at 72 h and at weeks 2, 3 and 4. (*n* = 3)	< 0.5 ng/µL DNA qPCR: no amplification product detected	0 CFU	Not performed	Not performed
Sampling tubes containing aerobic medium opened to environment during foetal sampling. Samples incubated at 37 °C and CFUs reported at 72 h and at weeks 2, 3 and 4. (*n* = 3)	< 0.5 ng/µL DNA qPCR: no amplification product detected	Not performed	0 CFU	Not performed
DNA spin columns: blank (no-template) controls from an Isolate II Genomic DNA kit (*n* = 2)	< 0.5 ng/µL DNA qPCR: no amplification product detected	Not performed	Not performed	152 reads assigned to 100 bacterial ESVs. Max read/ESV = 3

*CFU* colony forming unit, *ESV* exact sequence variant, *PBS* phosphate-buffered saline.

^a^Swabs incubated at 37 °C and CFUs reported at 72 h, then stored at 4 °C and CFUs reported for up to 4 weeks.

Potential airborne bacteria were passively sampled using open tubes and then incubated to determine if any viable, agar-cultivable microorganisms were present in the work area. No growth in aerobic or anaerobic media was observed after exposure. DNA extractions carried out from the media were below the detection limit (< 0.5 ng/µL of DNA), and no products were amplified by qPCR.

The dissection table surface and external surfaces of the amniotic sac and foetal intestinal compartments were swabbed to determine if microorganisms were present that could contaminate the foetal samples. The DNA extracted from all swabs was below the detection limit (< 0.5 ng/µL of DNA), and no products were amplified by qPCR. Anaerobic and aerobic liquid media were inoculated with the swabs and incubated but no growth was observed.

Two DNA spin columns from two Isolate II Genomic DNA kits (Bioline, Alexandria, Australia) were used as no-template controls and tested for background contamination using Illumina MiSeq next-generation sequencing. Collectively, bacterial reads in the no-template controls comprised 0.036% of the abundance of all community sequences in the GIT samples (860,749 bacterial sequences) and 0.071% of sequences assigned to the isolates cultured from ruminal fluid. These abundance values were used as a threshold for the two data sets to exclude sequences that were potentially present due to contamination as per Bokulich et al.^29^. After quality filtering, the two no-template controls contained 152 reads that were assigned to 100 bacterial exact sequence variants (ESVs). Seven of these ESVs were each assigned the highest number of contaminating reads which was 3. These 7 ESVs were identified as *Escherichia/Shigella* (4 ESVs), *Enterococcus* (2 ESVs) and *Mycoplasma* (1 ESV). The low read abundances and high level of singletons detected in controls suggest that sequencing cross-talk may be a likely source of reads present in no-template controls^30^.

The kits, environment and sampling procedures contributed minimally (less than 0.036% of total reads) or not at all to the community of microorganisms in the calf foetal GIT and amniotic fluid samples ruling out contamination as the source of observed microbial communities in the sample.

### Microbiome associated with the calf foetal GIT and amniotic fluid

To examine the spatial and temporal distribution of microbes of the calf foetal GIT, the maternal uterus was removed as a single unit 35–45 min after slaughter and ruminal fluid, ruminal tissue, caecal fluid, caecal tissue and meconium from the foetus, and amniotic fluid were collected. Samples underwent DNA extraction and analysis for microbial abundance and diversity using quantitative PCR (qPCR) and amplicon sequencing. Aerobic and anaerobic cultures were used to demonstrate a viable culturable fraction of the community in the ruminal fluid.

**Figure 1:**
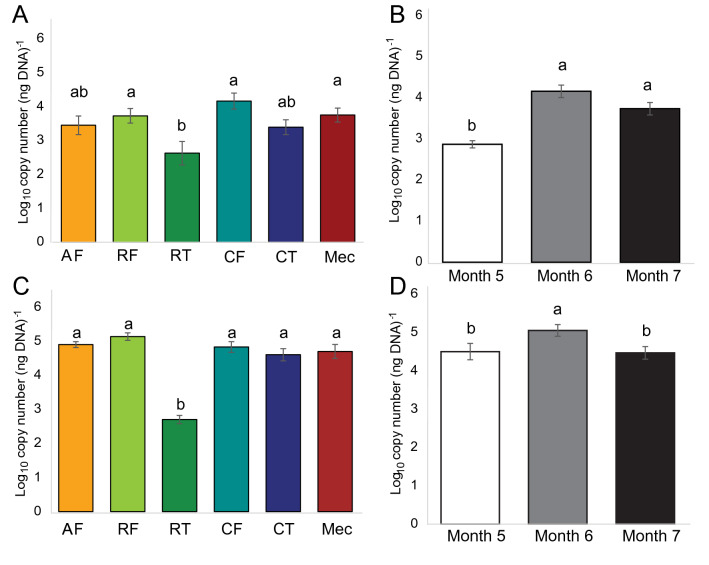
Quantitative PCR estimation (mean ± SE) of bacterial (**A**,**B**) and archaeal (**C**,**D**) 16S rRNA gene copy number in each GIT component and amniotic fluid (**A**,**C**) and at each gestational age (**B**,**D**). Amniotic fluid (AF), ruminal fluid (RF, n = 12), ruminal tissue (RT, n = 4), caecal fluid (CF, n = 8), caecal tissue (CT, n = 10), meconium (Mec, n = 11). Gestational age: 5 months (*n* = 16), 6 months (*n* = 18), 7 months (*n* = 18). Different letters above bars indicate a significant difference (*p* < 0.05) between means as determined via Tukey’s HSD and Dunn’s post hoc tests for bacterial and archaeal data, respectively.

Bacterial and archaeal 16S rRNA gene copy numbers were detected and quantified by qPCR in each GIT component and amniotic fluid of calf foetuses at each measured gestational age. The bacterial copy numbers were significantly lower in the ruminal tissues than in the ruminal fluid, caecal fluid and meconium (Fig. 1A). The bacterial 16S rRNA gene copy numbers were higher in the GIT and amniotic fluid of foetuses at 6 and 7 months gestation compared with those at 5 months (Fig. 1B). Archaeal copy numbers were significantly lower in the rumen tissues than in the other GIT components or amniotic fluid (Fig. 1C). The gene copy numbers for archaea were higher in the GIT and amniotic fluid of foetuses at 6 months than at 5 and 7 months (Fig. 1D).

To assess spatial and temporal differences in microbial taxa within the GIT and amniotic fluid, an in-depth community analysis was conducted. Illumina next-generation amplicon sequencing produced 860,749 bacterial sequences and 400,786 archaeal sequences. After quality filtering, a total of 559 bacterial and 1736 archaeal ESVs were identified. All bacterial ESVs and 50% of the archaeal ESVs had taxonomic assignments at the phylum level with confidences above 90%. At the genus level, 62% of bacterial and 50% of archaeal ESVs had taxonomic assignments with confidence levels > 90%. Rarefaction curves of total bacterial and archaeal ESVs per sample almost reached a plateau and species accumulation curves were saturated for all samples, suggesting that additional reads would have recovered very few additional ESVs (Supplementary Fig. S1).

The distribution of bacterial phyla across all samples was 32% Proteobacteria (more than 80% of which was assigned to the gamma-Proteobacteria), 31% Firmicutes and 26% Actinobacteria. The archaeal community was dominated by Euryarchaeota (88% of community reads) followed by Crenarchaeota and Korarchaeota with 6% and 5% of reads, respectively.

**Figure 2:**
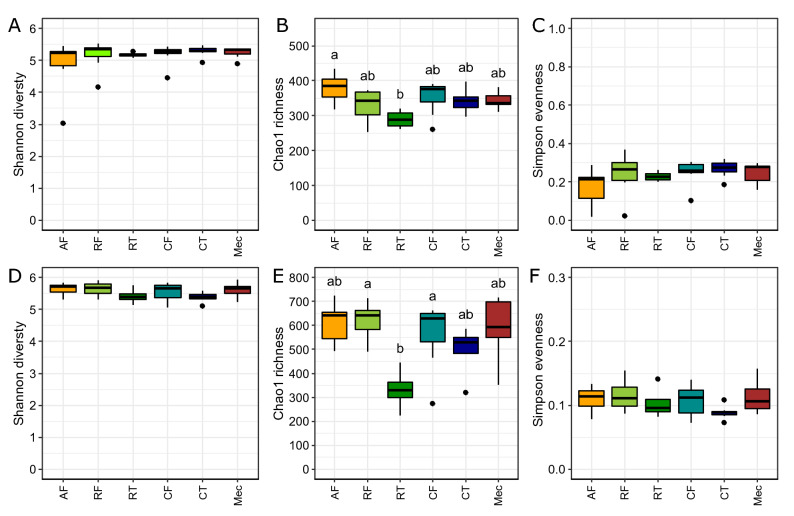
Box plots of bacteria (**A**–**C**) and archaea (**D**–**F**) for Shannon diversity indices (**A**,**D**), observed Chao1 estimates of ESV richness (**B**,**E**), and Simpson evenness (**C**,**F**) for communities between GIT components and amniotic fluid. Amniotic fluid (AF), ruminal fluid (RF), ruminal tissue (RT), caecal fluid (CF), caecal tissue (CT), meconium (Mec). Different letters above bars indicate a significant difference via Kruskal–Wallis testing (p < 0.05).

There were significant differences in Chao1 estimates of ESV richness between GIT compartments for both bacterial and archaeal communities (H_bacteria_ = 12.35, df = 5, p < 0.05, Fig. 2B; H_archaea_ = 16.92, df = 5, p < 0.05, Fig. 2E). Ruminal tissues had fewer bacterial ESVs than the amniotic fluid, and fewer archaeal ESVs than the ruminal and caecal fluid. We found no differences in community diversity (Shannon–Wiener) (Fig. 2A,D) or evenness (Simpsons evenness) between GIT compartments for bacteria or archaea (Fig. 2C,F).

There were significant differences in phylogenetic community structure between the GIT compartments for the bacterial communities using unweighted (i.e. presence absence transformed F_(5,32)_ = 5.13, p < 0.01) and weighted (F_(5,32)_ = 3.67, p < 0.01) UniFrac metrics, suggesting a turnover in dominant community members. In contrast, for archaeal phylogenetic community structure, the unweighted (F_(5,30)_ = 1.55, p < 0.01), but not weighted (F_(5,30)_ = 1.53, p > 0.05), UniFrac distance metrics suggest that differences between the GIT compartments are driven by rare community members.

**Figure 3:**
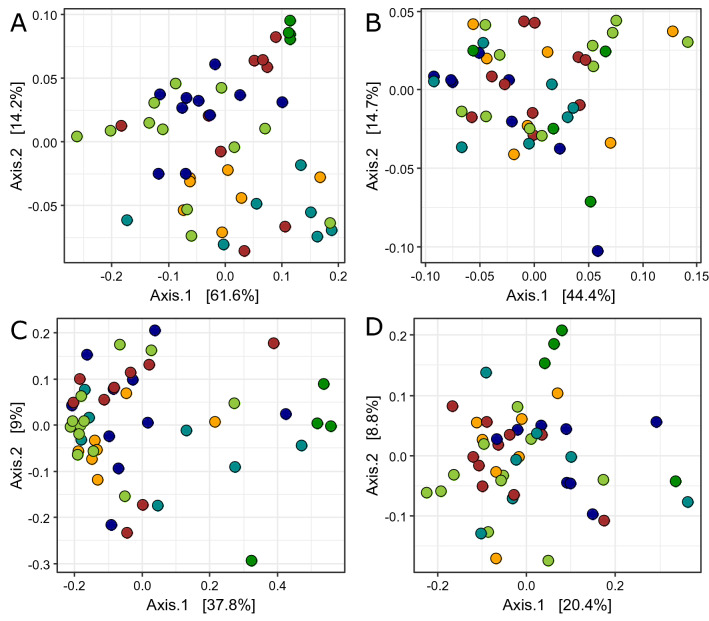
Non-metric multidimensional scaling ordination of bacterial (**A**,**C**) and archaeal (**B**,**D**) 16S rRNA profiles using weighted (**A**,**B**) and unweighted (**C**,**D**) UniFrac distance metrics. Colour is indicative of the sample type from which the community was sampled: orange  , amniotic fluid; light green  , ruminal fluid; dark green  , ruminal tissue; cyan  , caecal fluid; blue  , caecal tissue; red  , meconium. Two-dimension stress ranged from 0.06 to 0.08.

Principal coordinate analyses (PCoA) ordinations indicate that the significant differences are largely driven by ruminal tissue communities which cluster tightly for unweighted bacterial and archaeal community ordinations (Fig. 3). Weighted ordinations also indicate the presence of distinct bacterial communities between tissue and fluid components of the GIT as the caecal and ruminal tissue communities are distinct from their corresponding fluid communities (Fig. 3B). Unweighted bacterial (F_(2,32)_ = 2.91, p < 0.05), and archaeal (F_(2,30)_ = 1.55, p < 0.05), communities also exhibited significant phylogenetic grouping across gestational age, suggesting there was a turnover in community members with gestational age.

### Differential abundance analysis

**Figure 4:**
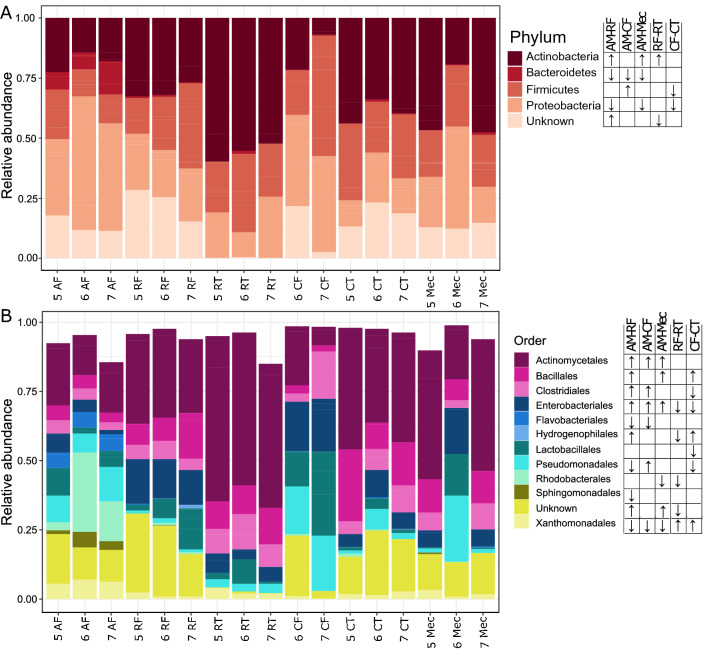
Mean relative abundance of bacterial phylum level (**A**) and order level (**B**) ESVs in each sample type (GIT component and amniotic fluid) at each gestational age (5, 6 and 7 months). Significant phylum-level and order-level changes in relative abundance between sample types are denoted next to the taxonomic key. For each pair of sample types, the arrow indicates the direction of change in abundance in the second sample type relative to the first. ESVs are clustered by colour to the levels of phylum and order. ESVs with taxonomic assignments at the phylum level and order level with confidence values lower than 90% are denoted as ‘Unknown’. Levels of gDNA in caecal fluid from foetuses at 5 months gestation were below the concentration threshold (0.5 ng/µL) for samples to be included in the sequencing run. Amniotic fluid (AF), ruminal fluid (RF), ruminal tissue (RT), caecal fluid (CF), caecal tissue (CT), meconium (Mec).

Differential abundance testing revealed that the dominant phyla in the amniotic fluid differed significantly (FDR adjusted *p* < 0.05) to those in the ruminal fluid, caecal fluid and meconium (Fig. 4; Supplementary Tables S1, S2 and S3). At the phylum level, the amniotic fluid was enriched for Bacteroidetes (order Flavobacteriales) compared with communities in the ruminal fluid, caecal fluid and meconium. The amniotic fluid was also enriched for Proteobacteria (orders Rhodobacterales, Xanthomonadales, Enterobacteriales, Sphingomonadales, Pseudomonadales) and diminished for Actinobacteria (order Actinomycetales) compared with communities in the ruminal fluid and meconium (Fig. 4A). The caecal fluid was also enriched for Firmicutes compared with communities in the amniotic fluid.

Significant phylum-level differences were also found between the fluid and tissues of the caecum and rumen across all ages (Fig. 4A, Supplementary Tables S4 and S5). Within the caecum, Firmicutes (predominantly driven by the order Lactobacillales) and Proteobacteria (orders Enterobacteriales and Pseudomonadales) were significantly enriched in the fluid compared with the tissues (Fig. 4A,B). Within the rumen, the phylum Actinobacteria was enriched in the tissues compared with the fluid (Fig. 4A).

Across all differential abundance tests, a total of 20 bacterial genera contributed the differential abundance signal. Of these 7 were also detected in the no-template controls. However, for each of these genera only a proportion of the contributing ESVs were present in controls with approximately %50 of reads assigned to ESVs that were not detected in controls (Supplementary Fig. S2).

**Figure 5:**
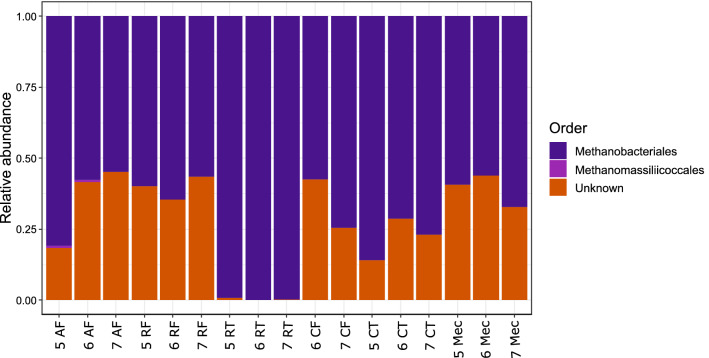
Mean relative abundance of archaeal ESVs in each sample type (GIT component and amniotic fluid) at each gestational age (5, 6 and 7 months). ESVs are clustered by colour to the level of order. ESVs with taxonomic assignments at the order level with confidence values lower than 90% are denoted as ‘Unknown’. The gDNA from caecal tissues at 5 months gestation was below the concentration threshold (0.5 ng/µL) for samples to be included in the sequencing run. Amniotic fluid (AF), ruminal fluid (RF), ruminal tissue (RT), caecal fluid (CF), caecal tissue (CT), meconium (Mec).

Differential abundance analysis was performed using only ESVs with high (> 90%) confidence values for phylum-level taxonomic assignments. All archaeal ESVs that satisfied this threshold were from the same phylum (Euryarchaeota) and encompassed two genera (*Methanobrevibacter* and *Methanomassiliicoccus*). Differential abundance analysis at the level of order revealed a significant enrichment of Methanobacteriales in ruminal tissues compared with all other GIT components and amniotic fluid (Fig. 5).

Culturing of Bacterial Isolates from Ruminal Fluid Samples.

Bacteria were cultured from the ruminal fluid of calf foetuses at 5, 6 and 7 months gestation. Ten visibly different colonies were isolated by sub-culturing up to 5 times. The ten isolated colonies were examined using the same NextGen sequencing pipeline as the original foetal samples.

Two of the ten isolated colonies (isolates 7 and 8) were not successfully sequenced. Data from the remaining 8 isolates indicated that the colonies were not pure cultures as each isolate contained reads assigned to between 68–169 ESVs. The taxonomic identification of the top 10 ESVs for each isolate are presented in (Supplementary Fig. S3). A total of 5 colonies (colonies 1, 2, 3, 5 and 6) contained reads that were largely associated with ESVs for the genera *Escherichia* or *Shigella*. The majority of reads from colony 9 were associated with ESVs identified as Stenotrophomonas and reads from colony 10 were associated with ESVs identified as *Brevibacterium*. Colony 4 contained reads mainly associated with ESVs from the genera *Enterococcus* but also had a number of reads associated with either *Escherichia* or *Shigella*. For the ESVs identified as belonging to cultured isolates, the number of corresponding reads in the combined bacterial no-template controls never exceeded 4. In contrast the top isolate ESVs were well represented in the GIT community collectively comprising ~ 30% of the relative abundance of any given GIT compartment (Supplementary Fig. S4), The isolate ESV reads were present in much greater abundances than the contribution from the kit controls, suggesting that sequences from the colony isolates were not due to kit contamination.

## Discussion

Studies reporting the presence of microorganisms in foetal or placental tissues are often received with scepticism when sample contamination is a possibility; such as when no kit controls are incorporated or when contamination sampling is limited^12,31^. The present study includes a complete set of contamination controls at different steps of the foetal sampling procedure and during sample preparation and analysis (Table 1). Culture media exposed to airborne contaminants during the sampling process had no CFUs after incubation, which suggests that the air was not a source of the agar-cultivable microorganisms. DNA extracted from all control swabs was below detection limits (< 0.5 ng/µL of DNA), and no products were amplified by qPCR, which supports that the swabbed locations were not a source of contaminating microorganisms in either the amplicon sequencing or culturing. The results for the foetal samples are therefore due to the presence of microorganisms in the amniotic fluid and foetal GIT present before birth and not due to sample contamination.

Bacterial ESVs for *Escherichia/Shigella*, Brevibacterium, *Enterococcus* and *Stenotrophomonas* were detected in the no-template kit controls. The number of reads in the combined bacterial no-template controls never exceeded 4 for a given ESV. In contrast the top ESVs identified as belonging to cultured isolates were assigned a minimum of 334 reads, with an average of 1388 reads, and were well represented in the GIT community collectively comprising ~ 30% of the relative abundance of any given GIT compartment. The total reads of archaea in the kits was insignificant with respect to the reads detected in the GIT samples. In several other studies, common contaminants of DNA extraction kits were *Sediminibacterium*, *Bradyrhizobiaceae*, *Methylobacterium* and *Propionibacterium*^32^. The current results allowed the subtraction of any potential contamination from the samples throughout the experiment.

Previous studies of infants at parturition have reported microbial communities in the placenta^14,16^, amniotic fluid^16,17^ and umbilical cord^18^ but spatial and temporal analyses of the GIT have not been reported. Microorganisms have been detected in the first meconium produced up to 2 days after birth in infants^20–22^ which prompted theories that gut colonisation might start prior to birth^14,16^. However, until now there has been no direct evidence. Our results show that the abundance of bacterial and archaeal communities in calf foetal fluids and tissues changed between 5- and 7-months gestation. At these gestational ages, the cervix is still tightly closed and thought to exclude bacteria from entering via the vagina. Our results indicate that initial colonisation of the GIT can occur before 5 months gestation, which suggests that microbial colonisation of the foetus is a developmental process. Distinct temporal and spatial microbial communities were identified in the foetal GIT and amniotic fluid during gestation. ESVs retrieved through metabarcoding of isolates cultured from the ruminal fluid were also observed in GIT communities, suggesting the cultured isolates were true members of the foetal calf GIT microbiome. To our knowledge this is the earliest reported detection of a foetus-associated microbial community and provides direct evidence that the GIT of the foetus contains a pioneer microbiome well before birth. The combination of culturing and molecular analysis, together with the rigorous use of controls, argues against the ‘sterile-foetus paradigm’ that is currently proposed^12^.

The foetal GIT components and amniotic fluid contained 559 bacterial and 1736 archaeal ESVs. Differences between the tissues and fluids in the different GIT compartments (rumen and caecum) indicate location-specific microbial selection is occurring. The phyla Proteobacteria (order Enterobacteriales) and Firmicutes (order Lactobacillales) were enriched dominant in the ruminal fluid, whereas the phylum Actinobacteria were enriched and dominant in ruminal tissue. Bacterial culturing confirmed that viable members of Proteobacteria, Actinobacteria and Firmicutes were present in the ruminal fluid. A compositionally similar community, comprising Proteobacteria, Firmicutes and Bacteroidetes, has been found to dominate the meconium and rumen of new-born calves^33–35^. In the ruminal fluid of mature cows, Firmicutes is the most abundant phylum, followed by Bacteroidetes^36^. The decrease of Proteobacteria in mature cows compared with calf foetuses and neonates is of interest due to the metabolic diversity associated with this group and its association with a variety of pathogens.

Caecal tissues supported a distinct microbial community across all ages compared with caecal fluid. The caecal tissues were dominated by Actinobacteria, whereas Firmicutes (order Lactobacillales) and Proteobacteria (order Enterobacteriales and Pseudomonadales) dominated the caecal fluid. This distribution is dissimilar to that in a study of 3-week-old calves, where Bacteroidetes was the dominant phylum in both caecal tissue and fluid^37^. This difference between pre and postnatal microbial communities of the caecum could be influenced by a variety of factors including the environment, nutrition and immune response.

The amniotic fluid harboured a distinct microbial community compared with the caecal fluid, ruminal fluid and meconium. Bacteroidetes were in highest abundance in the amniotic fluid but were present in low abundance in the ruminal fluid, caecal fluid or meconium. In contrast, Actinobacteria were enriched in the ruminal fluid whereas Firmicutes were enriched in the caecum fluid. These differences may reflect compartmental differences in the development of the ruminal, caecal and amniotic environments. In general, microbial communities in the caecum, and also the rumen, play a role in digestion via fermentation, although it is currently unknown whether the pioneer microbiome makes a metabolic contribution to the developing foetus.

Healthy bovine and human foetuses swallow up to 500 mL/day of amniotic fluid during the late second and third trimesters of gestation^38,39^. Meconium has been found in the amniotic fluid in normal pregnancies and could be related to the physiological development of the GIT with increasing gestational age. The commonality of microbial species in this study possibly reflects the cycling of material between compartments, meconium and amniotic fluid, whereas differences in their abundance suggests that some of these microbial species might begin to establish different physiological pathways depending on each compartment location, including between the fluids and tissues of the rumen and caecum. Based on our data, the events influencing the commonalities and the differences in microbial communities among the compartments of the GIT and amniotic fluid must have initiated before the foetuses reached 5 months gestation. Whether the circulation of amniotic microbes through the GIT has additional health benefits for the foetus (and/or perhaps the mother) remains to be investigated.

The archaeal phyla Euryarchaeota (*Methanobrevibacter* spp. and *Methanomassiliicoccus* spp.), Crenarchaeota and Korarchaeota associated with the foetal tissues are also present in the foetal calf GIT. Euryarchaeota comprise more than 99% of the ruminal and faecal communities at birth and in calves up to 2 weeks old^19,40,41^ and is the most abundant archaeal taxon in the human colon and mature cow rumen^42,43^.

The foetal and mature bovine GIT share a number of bacterial and archaeal species with mature cows including *Lactobacillales* spp., *Propionibacterium* spp., *Acinetobacter* spp., *Escherichia* or *Shigella* spp., *Streptococcus* spp.^36,44^, *Methanobrevibacter* spp. and *Methanomassiliicoccus* spp.^45^. We speculate that the presence of bacterial and archaeal communities in the foetus could have been introduced by translocation from the GIT of the mother to the foetal GIT and amniotic fluid. Although the origin of pioneer microbiota in the foetal GIT and amniotic fluid is unknown, it could be derived from the mother’s GIT epithelium, which is a rich source of diverse microbial communities^46^.

Novel clades of bacteria and archaea are likely to be involved in the early colonisation of the foetal GIT and amniotic fluid. At the genus level, 62% of bacterial and 50% of archaeal ESVs had taxonomic assignments with confidence levels > 90%, therefore it is likely that 38% of bacterial ESVs and 50% of archaeal ESVs represent species that are yet to be represented in sequence read databases. For the ill-defined bacterial ESVs, the lowest confidence level for family-level assignment was 99% indicating that these ESVs are likely to be closely related to known taxa and do not represent entirely novel clades. For the ill-defined archaeal ESVs, less than 1% were assigned to the archaeal domain with confidence (> 97%). All ESVs from this 1% were identified as belonging to the order Methanomassiliicoccales. For the remaining 99% (approximately 806 ESVs), the greatest confidence to which they could be assigned to the archaeal domain was 61%, suggesting that these ESVs may represent novel clades hitherto undocumented.

While the contribution of contaminants from kits and reagents examined in the present study allowed us to exclude with reasonable confidence that the detected taxa originated from external sources, we acknowledge that our study has several technical limitations. Specifically, study resources limited the investigation to analyses of only two no-template controls. The aerobic and anaerobic media used in this study are commonly used in the authors’ laboratory to grow GIT microorganisms. However, to test and confirm that ‘no growth’ is not due to an inability of the media to support growth under the cultivation conditions, a suitable positive control microorganism grown alongside the test plates for contamination samples should be included in future studies. This study was designed to detect low concentrations of contaminating microorganisms, but for further understanding of the quantities of microorganisms in contamination control samples and in the test samples, future studies could use a qPCR method with a limit of quantitation lower than 0.5 ng/µL of DNA. Overall, future studies of microorganisms in the foetal GIT will contribute to refine our understanding of the role of contamination, and we envisage that future investigations should include a well-designed protocol to monitor each step in the procedure where microbial contamination is possible. Such studies could increase contaminant detection and have greater statistical power if several no-template controls from each kit were analysed. This would address valid criticism of the lack of contamination controls associated with other studies of foetal GIT colonisation.

Until recently the foetal GIT was considered to be a completely sterile environment. The universal acceptance of such a major paradigm shift will require multiple lines of supporting evidence. Although we have taken a conservative approach to metabarcoding analysis, to avoid premature or over-extrapolated mechanistic hypotheses, our manuscript is a significant contribution to this growing body of work as our data demonstrates that there are large repeatable phylogenetic differences between GIT compartments. This clearly demonstrates the presence of a foetal microbiome. If the observed microbial reads were due to source or kit contamination the repeatability of the microbiome changes between GIT compartments would have to be due to chance, which is highly unlikely. Our manuscript also shows that there is differentiation between microbial communities from GIT compartments which is suggestive of directed selection and the formation of functionally distinct communities which may be critical to the development of the mature GIT.

In summary, our findings provide the first direct evidence that microbial communities exist in the foetal GIT and amniotic fluid. The kits, environment and sampling procedures contributed minimally or not at all to the community of microorganisms in the calf foetal GIT and amniotic fluid. The presence of microbial communities in the GIT and amniotic fluid of foetuses at 5 months gestation is much earlier than previously proposed. The microbial communities differed in their composition and abundance between rumen, caecum, meconium and amniotic fluid, and differed between ruminal and caecal fluids and their associated tissues. Further investigation is required to determine the path of initial colonisation of the pioneer microbiome, whether these microbial communities are involved in metabolic interactions with the foetus, and whether they are involved in priming foetal immunity.

## Materials and methods

### Experimental design and sample collection

This study was carried out in accordance with the provisions in the Australian Code of Practice for the Care and Use of Animals for Scientific Purposes (7th edition, 2004) and all protocols were approved by the Animal Ethics Committee of La Trobe University. Twelve Angus × Friesian cattle foetuses at 5, 6 and 7 months gestation (*n* = 4 per age) were collected from Radford Warragul Abattoir, Victoria, Australia. Approximately 35–45 min after cows were slaughtered by abattoir staff, the intact uterus (containing the placenta and foetus) was removed. All sampling was conducted at the abattoir using sterile equipment and procedures. The outside surface of the amniotic sac was rinsed three times with sterilised phosphate-buffered saline (PBS; pH 7.0) to remove excess blood. The amnion was cut using sterile scalpels and amniotic fluid was sampled. The amniotic fluid was suctioned using sterile 50-mL syringes with tubing and the amniotic fluid was transferred immediately into 50-mL tubes. Then, the amniotic sac was opened further, the umbilical cord was cut, and the foetus was removed. The abdomen of the foetus was opened using sterilised equipment and the rostral and caudal ends of each GIT compartment were tied with sterile surgical thread to avoid mixing of the contents. The compartments were then separated between the ties. Each compartment was longitudinally incised along the dorsal line. Tissue samples (~ 2 cm^2^) of the rumen were taken from the dorsal area and caecal tissue samples were taken from the region 5 cm after the ileocaecal valve. Meconium pellets (~ 100 g) were taken by severing the rectum 5 cm from the anus. The fluid, tissue and meconium samples were collected into sterile 15-mL or 50-mL polypropylene centrifuge tubes. All samples were immediately placed into dry ice for transport. All samples were processed within 6 h of collection to extract gDNA.

### DNA extraction

Genomic DNA was extracted from 250 mg of ruminal tissue, ruminal fluid, caecal tissue, caecal fluid and meconium. An 8-mL aliquot of amniotic fluid was centrifuged (11,000*g*, 5 min) to produce sufficient material in the pellet for extraction. DNA was extracted using an Isolate II Genomic DNA kit following the manufacturer’s instructions. Final DNA concentrations and purity were estimated using a P330-Class NanoPhotometer (Implen, München, Germany). All samples were stored at − 80 °C for later analysis.

### 16S rRNA library preparation and sequencing

Libraries were prepared for sequencing on an Illumina MiSeq following the protocol ‘16S Metagenomic Sequencing Library Preparation’ (Part # 15044223 Rev. B; Illumina, San Diego, CA, USA). The locus-specific primers were the universal 16S rRNA primer pairs S-D-Bact-0341-b-S-17 (5′-CCTACGGGNGGCWGCAG-3′) and S-D-Bact-0785-a-A-21 (5′-GACTACHVGGGTATCTAATCC-3′), Archaea349F (5′-GYGCASCAGKCGMGAAW-3′), and Archaea806R (5′-GGACTACVSGGGTATCTAAT-3′), which target the V3–V4 region of the bacterial and archaeal 16S rRNA genes, respectively. Primers had forward (5′-TCGTCGGCAGCGTCAGATGTGTATAAGAGACAG-3′) and reverse (5′-GTCTCGTGGGCTCGGAGATGTGTATAAGAGACAG-3′) Illumina overhang adaptors merged to the 5′ ends.

PCR was performed in 25-µL reactions using 5 µL of each forward and reverse primer (10 µM), 12.5 µL 2 × KAPA HiFi HotStart ReadyMix (Kapa Biosystems, Boston, MA, USA) and 2.5 µL of genomic DNA template (5 µL/ng). PCR cycle settings for the amplification of the bacterial and archaeal V3–V4 region were as follows: denaturation at 95 °C for 3 min, followed by 28 (bacterial) or 30 (archaeal) cycles of 30 s at 95 °C, 30 s at 55 °C and 30 s at 72 °C, followed by an extension step at 72 °C for 5 min. To normalise libraries prior to pooling, the DNA content of PCR reactions was quantified using an Agilent D1000 ScreenTape System (Agilent Technologies, CA, USA). Samples were adjusted to the same molarity (4 nM), pooled, and paired-end sequenced (2 × 300 bp) on an Illumina MiSeq platform. The MiSeq run was performed at La Trobe University Genomics Platform (Melbourne, Australia).

### Analysis of sequence data

Raw, de-multiplexed, fastq files were re-barcoded, joined and quality filtered using the UPARSE clustering pipeline (USEARCH version 9.2.64; https://drive5.com/uparse)^46^. Paired-end reads were merged such that alignments with > 20 bp difference (i.e. approximately more than 10–14% mismatched) were discarded, and merged reads less than 300 bp in length were discarded. Reads that could not be assembled were discarded. Merged reads were quality filtered by discarding reads with total expected errors > 1.0. ESVs were generated with the “unoise3” command^47^. Taxonomic assignments were performed using the UTAX algorithm. Reference databases were created using the RDP_trainset_15 dataset, available from the UTAX downloads page (https://drive5.com/usearch/manual/utax_downloads.html). A minimum percentage identity of 90% was required for an ESV to be considered a database match hit. ESVs identified as chloroplasts and mitochondrial DNA were removed from the data. After filtering, the average read number (± SD) for each git compartment was: 4342 ± 1594 reads for the AM , 17,276 ± 17,376 reads for the CF, 6182 ± 3918 reads for the CT, 5732 ± 3262 reads for the Mec, 5630 ± 2234 reads for the RF and 5847 ± 4052 reads for the RT. The rarefying threshold of 1000 reads was chosen to maximise the amount of reads included in the analysis whilst minimize the number of samples excluded from the analysis. A total of three bacterial samples resulted in reads below the rarefication threshold (1000 reads) and were excluded from downstream alpha- and beta-diversity analyses. The samples were: Month_5_Mec-2, Month_6_CF-3 and Month_6_Mec-1. DNA extraction or library preparation was unsuccessful for the following samples: n = 0 (5 months cecum fluid), n = 1 (6 and 7 months rumen tissue, 7 months amniotic fluid), n = 2 (5 months rumen tissue, 6 months amniotic fluid), n = 3 (5 and 6 months cecum tissue, 5 months meconium), the remaining GIT compartments and months were n = 4. Raw fastq files for this project and metadata have been deposited with the NCBI SRA database and can be accessed using Bioproject ID: PRJNA421384 or SRA study ID: SRP126299.

### Bacterial culture from ruminal fluid samples and identification of bacterial isolates

A 50-mL sample of ruminal fluid was taken from each foetus and maintained under anaerobic conditions (Oxoid AnaeroJar with an AnaeroGen™). A 1-mL aliquot of the ruminal fluid was transferred to anaerobic solid medium and cultured at 37 °C for 48–72 h. The anaerobic solid medium had the following composition (per litre of distilled water): 15 g agar (Oxoid), 10 g peptone (Oxoid), 10 g yeast extract, 8.8 g Oxoid Lab-Lemco beef extract powder, 10 g proteose peptone (Oxoid), 12 g dextrose, 10 g KH_2_PO_4_, 12 g NaCl, 20 g soluble starch, 1.2 g l-cysteine hydrochloride and 0.3 g sodium thioglycollate with a pH (at 25 °C) of 7.3 ± 0.1. Colonies were isolated and subcultured 5 times onto new agar media plates, except for the control plates (*n* = 3) which showed no microbial growth. Colonies were subcultured on fresh media and DNA extracted. The extracts for 5, 6 and 7 months were combined prior to next-generation sequencing of the 16S rRNA genes to characterise the taxonomic structure.

### Quantitative PCR

Quantitative PCR (qPCR) was used to enumerate total bacterial and archaeal DNA copy number in each sample type (GIT component and amniotic fluid) as an indicator of abundance. The primer pairs bacF (5′-CCATTGTAGCACGTGTGTAGCC-3′) and bacR (5′-CGGCAACGAGCGCAACCC-3′) were used to amplify bacterial 16S rRNA, and Archaea364F (5′-CCTACGGGRBGCAGCAGG-3′) and Archaea1386R (5′-GCGGTGTGTGCAAGGAGC-3′) were used to amplify archaeal 16S rRNA. PCR reactions were run in triplicate on a CFX Connect Real-Time PCR Detection System (Bio-Rad, CA, USA). The total volume of each reaction mix was 20 μL, comprising 10 μL of SensiFAST SYBR Green Master Mix (Bioline), 0.4 μL of each forward and reverse primer (10 µM), sterile DNA-free water, and 7 ng of DNA. Triplicate control samples (no-DNA templates) were included to verify that no contaminating nucleic acid was introduced into the master mix or into samples. Positive controls contained gDNA extracted from laboratory cultured bacteria (*E. coli* strain DH5α) and archaea (*Methanobrevibacter smithii*)*,* respectively. Thermocycling conditions were as follows: initial denaturation for 3 min at 94 °C, followed by 40 cycles of 10 s at 94 °C, 30 s at 60 °C. This was followed by a dissociation protocol (increasing 1 °C every 30 s from 60 °C to 98 °C).

A standard curve was constructed using serial tenfold dilutions from 10^−1^ to 10^−11^ of DNA from the bacterium *E. coli* strain DH5α (Stratagene, CA, USA) or the archaeon *M. smithii.* Real-time PCR efficiency ranged from 97 to 102%. Copy numbers for each standard curve were calculated based on the following equation: (N_A_ × A × 10^−9^)/(660 × *n*), where N_A_ is the Avogadro constant (6.02 × 10^23^ mol^−1^), A is the molecular weight of DNA molecules (ng/mol) and *n* is the length of amplicon (bp).

### Control procedures for sample contamination

Potential airborne bacteria were passively sampled to determine if there was a detectable contribution of environmental bacteria contaminating the foetal samples. Sampling tubes containing aerobic or anaerobic medium were opened and exposed to the dissection area in the abattoir for the duration of sampling from each foetus. The exposed media were incubated at 37 °C and samples taken at 72 h and at 2, 3 and 4 weeks. DNA was extracted using an Isolate II Genomic DNA kit. Final DNA concentrations and purity were estimated using a P330-Class NanoPhotometer.

The dissection table and external surfaces of the amniotic sac and intestinal compartments were swabbed to determine if there was a detectable contribution of bacteria contaminating the foetal samples. For each foetus, 9 swabs were taken at six locations using sterile Fisherbrand synthetic-tipped applicator swabs (Thermo Fisher Scientific, MA, USA). The surface of the dissection table (first location) was washed with 70% ethanol and then swabbed prior to dissecting each foetus. The external surface of the amniotic sac (second) was rinsed three times with sterilised PBS to remove excess blood and then swabbed prior to opening the sac. The skin of the foetal abdomen (third) was rinsed three times with sterilised PBS to remove amniotic fluid and then swabbed prior to opening. The external (mesenteric) surfaces of the rumen (fourth), caecum (fifth) and rectum (sixth location) were separately swabbed prior to opening. The 9 swabs for each foetus were tested for the presence of microorganisms, using three swabs for each of the three methods: qPCR, anaerobic culture and aerobic culture. DNA was extracted from three of the swabs using an Isolate II Genomic DNA kit. Final DNA concentrations and purity were estimated using a P330-Class NanoPhotometer. Quantitative PCR was used to more accurately quantify the presence of DNA.

Anaerobic and aerobic liquid mediums were inoculated from three swabs each. Three swabs were placed into a 2.5-L anaerobic Oxoid AnaeroJar with an AnaeroGen sachet (Thermo Fisher Scientific) and three swabs were placed into aerobic Oxoid Nutrient Broth (Thermo Fisher Scientific). All mediums were incubated at 37 °C for 48–72 h. Monitoring for growth during storage at 4 °C was continued for up to one month. The anaerobic liquid medium had the following composition (per litre of distilled water): 10 g peptone (Oxoid), 10 g yeast extract, 8.8 g Oxoid Lab-Lemco beef extract powder, 10 g proteose peptone (Oxoid), 12 g dextrose, 10 g KH_2_PO_4_, 12 g NaCl, 20 g soluble starch, 1.2 g L-cysteine hydrochloride and 0.3 g sodium thioglycollate with a pH (at 25 °C) of 7.3 ± 0.1. The aerobic nutrient broth had the following composition (per litre of distilled water): 10 g Lab-Lemco powder, 10 g peptone and 5 g NaCl, with a pH (at 25 °C) of 7.5 ± 0.2.

### Controls for kit contamination

Two blank DNA spin columns from two different Isolate II Genomic DNA kits were used as no-template controls to determine if the kits were a source of contamination during DNA extraction and library preparation of samples. The no-template controls were tested using qPCR and Illumina MiSeq next-generation sequencing.

### Statistical analysis of the total community

The adequacy of the sampling effort to capture the microbial community richness was examined by generating species rarefaction curves and species accumulation plots using the ‘rarecurve’ and ‘specaccum’ functions in the R library vegan (v. 2.3-4)^48^ using R 3.2.0^49^. Alpha- and beta-diversity analyses were performed on archaeal and bacterial OTU-matrices rarefied to depths of 2000 and 1000 reads, respectively, using the ‘phyloseq’ (v. 1.16-2)^50^ and ‘vegan’ (v. 2.4-0)^48,51^ packages in the R programming language (v. 3.3.1)^49^. Normality and variance homogeneity of the data were tested using the ‘shapiro.test’ and ‘bartlett.test’ functions. As normality and homogeneity of variance assumptions were not met, Kruskal–Wallis tests were carried out using the ‘kruskal.test’ function with Dunn’s test of multiple comparisons used for post hoc testing. The NMDS ordinations were used to visualise differences between communities from different sample types (GIT components and amniotic fluid). Dissimilarity matrices were generated using the weighted and unweighted UniFrac metrics^52^. Analysis of similarity (ANOSIM) procedures were implemented to test for significant differences in the mean group centroids^53^. Differential abundance testing of ESVs was performed using the DESeq2 extension available within the ‘phyloseq’ package^50,54^. Tests were performed by applying model-fitting normalisation to unrarefied ESV tables as recommended by McMurdie and Holmes for each taxonomic rank^50^. For differential abundance tests only ESVs with high (> 90%) confidence values for phylum-level taxonomic assignments were considered. All low confidence taxonomic assignment we re-classified as ‘unknown’. Differences were considered significant if Benjamin-Hochberg adjusted p < 0.05. The confidence values for taxonomic assignments of differentially abundant ESVs are listed in supplementary tables.

### Statistical analysis of bacterial culture and identification from ruminal fluid samples

gDNA from cultures of ruminal fluid were sequenced alongside community samples on an Illumina MiSeq. Filtering and quality control of isolate sequence data was identical to the process used for community samples. ESVs that contributed < 0.07% to the set of reads associated with cultured isolates were excluded from the final analysis.

### Statistical analysis of the quantitative PCR

Means were compared using a two-way factorial ANOVA with post hoc multiple comparisons using Tukey’s honest significant difference test. Due to the unbalanced design, type II sums of squares were calculated using the ‘Anova’ function from the R package ‘car’^55^.

## Supplementary information

Supplementary Information.
