# Supplementary material for: A pioneer calf foetus microbiome
Source: Sci Rep. 2020 Oct 19;10:17712. doi: 10.1038/s41598-020-74677-7 (PMC7572361; doi:10.1038/s41598-020-74677-7)
Supplement: Supplementary file 1 — Supplementary Information. [file 41598_2020_74677_MOESM1_ESM.docx]

**Title:** **A Pioneer Calf Foetus Microbiome**

Cesar E. Guzman^1^, Jennifer L. Wood^1,2,6^, Eleonora Egidi^1,2,5^, Alison C. White-Monsant^3^, Lucie Semenec^1^, Sylvia V.H. Grommen^1^, Elisa L. Hill-Yardin^4^, Bert De Groef^1^, Ashley E. Franks^1,2*^

^1^ Department of Physiology, Anatomy and Microbiology, La Trobe University, Melbourne, Victoria 3086, Australia.

^2^ Centre for Future Landscapes, La Trobe University, Melbourne, Victoria 3086, Australia.

^3^ Department of Animal, Plant and Soil Sciences, Centre for Agribiosciences, La Trobe University, Melbourne, Victoria 3086, Australia.

^4^ School of Health and Biomedical Sciences, RMIT University, Clements Drive, Bundoora, Victoria 3083, Australia.

^5^ Hawkesbury Institute for the Environment, Western Sydney University, Richmond, NSW 2753, Australia.

^6^ Faculty of Science, Engineering and Technology, Swinburne University of Technology, Hawthorn, VIC 3122, Australia.

^*^ Corresponding author: A.Franks@latrobe.edu.au

**Supplementary figures and tables**

**Supplementary Figure S1.** Rarefaction and species accumulation curves of bacterial (A, B) and archaeal (C, D) OTUs per sample to rarefaction depths of 1000 and 2000 reads, respectively.

**Supplementary Figure S2.** Genus-level distribution of reads from genera determined to be significantly differential abundant between various GIT compartments (see Supplemental tables S1 - S5) across all GIT compartments and no-template controls. Reads from genera identified as contributing to the controls have been separated into ESVs present in controls (denoted with ‘_C’) and ESVs not detected in controls.

**Supplementary Figure S3.** Genus-level distribution of top 10 most abundant ESVs from each cultured isolate (45 ESVs total).

**Supplementary Figure S4.** Distribution of the top 10 most abundant ESVs from each cultured isolate (45 ESVs total) across the GIT communities.

**Supplementary Table S1.** Taxa identified as significantly differentially (Benjamin–Hochberg adjusted *p* < 0.05) abundant between amniotic fluid and meconium.

| BM | L2FC | L2FC SE | *P* | Phylum | Class | Order | Family | Genus |
| --- | --- | --- | --- | --- | --- | --- | --- | --- |
| Enriched in amniotic fluid | | |  |  |  |  |  |  |
| 78.48 | -3.26 | 1.34 | 0.03 | Bacteroidetes (1.0) |  |  |  |  |
| 163.08 | -3.56 | 1.38 | 0.04 | Firmicutes | Bacilli | Lactobacillales | Streptococcaceae | Streptococcus (1.0) |
| 1543.04 | -1.40 | 0.53 | 0.02 | Proteobacteria (1.0) |  |  |  |  |
| 100.66 | -6.87 | 2.04 | 0.01 | Proteobacteria | Alphaproteobacteria | Rhodobacterales (1.0) |  |  |
| 126.03 | -6.52 | 2.04 | 7E-03 | Proteobacteria | Alphaproteobacteria | Rhodobacterales | Rhodobacteraceae | Paracoccus (0.9) |
| 77.82 | -1.42 | 0.49 | 0.01 | Proteobacteria | Gammaproteobacteria | Xanthomonadales (1.0) |  |  |
| 59.80 | -9.12 | 2.07 | 1E-04 | Proteobacteria | Gammaproteobacteria | Enterobacteriales | Enterobacteriaceae | Buttiauxella (0.48) |
| 82.83 | -1.76 | 0.48 | 2E-03 | Proteobacteria | Gammaproteobacteria | Xanthomonadales | Xanthomonadaceae | Stenotrophomonas (0.99) |
| Enriched in meconium | | |  |  |  |  |  |  |
| 1243.30 | 1.14 | 0.36 | 0.01 | Actinobacteria(1.0) |  |  |  |  |
| 1304.06 | 2.08 | 0.38 | 7E-07 | Actinobacteria | Actinobacteria | Actinomycetales (1.0) |  |  |
| 97.88 | 1.88 | 0.41 | 1E-04 | Actinobacteria | Actinobacteria | Actinomycetales | Corynebacteriaceae | Corynebacterium (0.99) |
| 160.35 | 1.42 | 0.39 | 2E-03 | Actinobacteria | Actinobacteria | Actinomycetales | Nocardiaceae | Gordonia (0.11) |
| 987.43 | 1.73 | 0.32 | 4E-06 | Actinobacteria | Actinobacteria | Actinomycetales | Propionibacteriaceae | Propionibacterium (1.0) |
| 498.21 | 2.03 | 0.59 | 5E-03 | Firmicutes | Bacilli | Bacillales (1.0) |  |  |
| 422.96 | 1.85 | 0.49 | 2E-03 | Firmicutes | Bacilli | Bacillales | Staphylococcaceae | Staphylococcus (1.0) |
| 171.65 | 1.76 | 0.63 | 0.02 | Firmicutes | Clostridia | Clostridiales | Clostridiales Incertae Sedis_XI | Anaerococcus (1.0) |
| 1267.42 | 2.32 | 0.80 | 0.01 | Proteobacteria | Gammaproteobacteria | Enterobacteriales (1.0) |  |  |
| 839.66 | 2.49 | 0.76 | 6E-03 | Proteobacteria | Gammaproteobacteria | Enterobacteriales | Enterobacteriaceae | Escherichia/Shigella (0.94) |

L2FC, log twofold change in abundance; BM, mean abundance (base mean). Average confidence of the taxonomical assignment is indicated in parentheses.

**Supplementary Table S2.** Taxa identified as significantly differentially (Benjamin–Hochberg adjusted *p* < 0.05) abundant between amniotic fluid and caecal fluid.

| BM | L2FC | L2FC SE | *P* | Phylum | Class | Order | Family | Genus |
| --- | --- | --- | --- | --- | --- | --- | --- | --- |
| Enriched in amniotic fluid | | |  |  |  |  |  |  |
| 78.48 | -3.53 | 1.49 | 0.04 | Bacteroidetes (1.0) | |  |  |  |
| 27.80 | -5.02 | 1.88 | 0.02 | Bacteroidetes | Flavobacteriia | Flavobacteriales (1.0) | |  |
| 77.82 | -2.53 | 0.55 | 1E-04 | Proteobacteria | Gammaproteobacteria | Xanthomonadales (1.0) | |  |
| 82.83 | -2.52 | 0.54 | 1E-04 | Proteobacteria | Gammaproteobacteria | Xanthomonadales | Xanthomonadaceae | Stenotrophomonas (0.99) |
| Enriched in caecal fluid | | |  |  |  |  |  |  |
| 1304.06 | 1.41 | 0.42 | 3E-03 | Actinobacteria | Actinobacteria | Actinomycetales (1.0) | |  |
| 97.88 | 1.25 | 0.46 | 7E-03 | Actinobacteria | Actinobacteria | Actinomycetales | Corynebacteriaceae | Corynebacterium |
| 987.43 | 1.37 | 0.36 | 1E-03 | Actinobacteria | Actinobacteria | Actinomycetales | Propionibacteriaceae | Propionibacterium (1.0) |
| 1394.36 | 2.00 | 0.57 | 2E-03 | Firmicutes (1.0) | |  |  |  |
| 371.03 | 3.16 | 0.70 | 1E-04 | Firmicutes | Clostridia | Clostridiales (1.0) | |  |
| 171.65 | 2.88 | 0.69 | 4E-04 | Firmicutes | Clostridia | Clostridiales | Clostridiales_Incertae_Sedis_XI | Anaerococcus (1.0) |
| 73.56 | 2.61 | 0.90 | 4E-03 | Firmicutes | Clostridia | Clostridiales | Clostridiales_Incertae_Sedis_XI | Finegoldia |
| 1267.42 | 3.38 | 0.89 | 1E-03 | Proteobacteria | Gammaproteobacteria | Enterobacteriales (1.0) | |  |
| 839.66 | 3.85 | 0.85 | 1E-04 | Proteobacteria | Gammaproteobacteria | Enterobacteriales | Enterobacteriaceae | Escherichia/Shigella (0.94) |
| 454.15 | 2.92 | 0.83 | 2E-03 | Proteobacteria | Gammaproteobacteria | Pseudomonadales (1.0) | |  |
| 436.92 | 3.15 | 0.84 | 1E-03 | Proteobacteria | Gammaproteobacteria | Pseudomonadales | Moraxellaceae | Acinetobacter (1.0) |

L2FC, log twofold change in abundance; BM, mean abundance (base mean). Average confidence of the taxonomical assignment is indicated in parentheses.

**Supplementary Table S3.** Taxa identified as significantly differentially (Benjamin–Hochberg adjusted *p* < 0.05) abundant between amniotic fluid and ruminal fluid.

| BM | L2FC | L2FC SE | *P* | Phylum | Class | Order | Family | Genus |
| --- | --- | --- | --- | --- | --- | --- | --- | --- |
| Enriched in amniotic fluid | | |  |  |  |  |  |  |
| 78.48 | -4.06 | 1.29 | 2E-03 | Bacteroidetes (1.0) |  |  |  |  |
| 27.80 | -4.69 | 1.63 | 0.01 | Bacteroidetes | Flavobacteriia | Flavobacteriales (1.0) | |  |
| 25.17 | -5.19 | 1.78 | 0.01 | Bacteroidetes | Flavobacteriia | Flavobacteriales | Flavobacteriaceae | Chryseobacterium (0.99) |
| 1543.04 | -1.16 | 0.51 | 0.02 | Proteobacteria (1.0) |  |  |  |  |
| 20.14 | -6.19 | 2.17 | 0.01 | Proteobacteria | Alphaproteobacteria | Sphingomonadales (0.99) | |  |
| 25.55 | -6.33 | 2.32 | 0.02 | Proteobacteria | Alphaproteobacteria | Sphingomonadales | Sphingomonadaceae | Novosphingobium (0.41) |
| 454.15 | -1.73 | 0.73 | 0.03 | Proteobacteria | Gammaproteobacteria | Pseudomonadales (1.0) | |  |
| 436.92 | -2.49 | 0.74 | 3E-03 | Proteobacteria | Gammaproteobacteria | Pseudomonadales | Moraxellaceae | Acinetobacter (1.0) |
| 77.82 | -1.60 | 0.47 | 2E-03 | Proteobacteria | Gammaproteobacteria | Xanthomonadales (1.0) | |  |
| 82.83 | -1.90 | 0.46 | 2E-04 | Proteobacteria | Gammaproteobacteria | Xanthomonadales | Xanthomonadaceae | Stenotrophomonas (0.99) |
| Enriched in ruminal fluid | | |  |  |  |  |  |  |
| 1243.30 | 0.78 | 0.35 | 0.02 | Actinobacteria (1.0) |  |  |  |  |
| 1304.06 | 1.95 | 0.37 | 7E-07 | Actinobacteria | Actinobacteria | Actinomycetales (1.0) |  |  |
| 97.88 | 1.20 | 0.40 | 8E-03 | Actinobacteria | Actinobacteria | Actinomycetales | Corynebacteriaceae | Corynebacterium (0.99) |
| 160.35 | 1.55 | 0.37 | 2E-04 | Actinobacteria | Actinobacteria | Actinomycetales | Nocardiaceae | Gordonia (0.11) |
| 987.43 | 1.74 | 0.31 | 1E-06 | Actinobacteria | Actinobacteria | Actinomycetales | Propionibacteriaceae | Propionibacterium (1.0) |
| 498.21 | 2.41 | 0.57 | 8E-05 | Firmicutes | Bacilli | Bacillales (1.0) |  |  |
| 11.25 | 18.07 | 5.11 | 2E-03 | Firmicutes | Bacilli | Bacillales | Bacillaceae_1 | Bacillus (0.87) |
| 25.65 | 18.65 | 5.11 | 1E-03 | Firmicutes | Bacilli | Bacillales | Bacillales_Incertae_Sedis_X | Thermicanus (1.0) |
| 422.96 | 1.71 | 0.48 | 1E-03 | Firmicutes | Bacilli | Bacillales | Staphylococcaceae | Staphylococcus (1.0) |
| 13.27 | 7.45 | 3.14 | 0.05 | Firmicutes | Bacilli | Lactobacillales | Carnobacteriaceae | Atopostipes (0.97) |
| 371.03 | 1.56 | 0.61 | 0.02 | Firmicutes | Clostridia | Clostridiales (1.0) |  |  |
| 171.65 | 2.31 | 0.60 | 7E-04 | Firmicutes | Clostridia | Clostridiales | Clostridiales_Incertae_Sedis_XI | Anaerococcus (1.0) |
| 7.02 | 21.90 | 5.09 | 8E-07 | Proteobacteria | Betaproteobacteria | Hydrogenophilales (1.0) | |  |
| 5.63 | 21.12 | 5.09 | 2E-04 | Proteobacteria | Betaproteobacteria | Hydrogenophilales | Hydrogenophilaceae | Tepidiphilus (1.0) |
| 1267.42 | 4.38 | 0.78 | 3E-07 | Proteobacteria | Gammaproteobacteria | Enterobacteriales (1.0) | |  |
| 839.66 | 3.69 | 0.75 | 2E-05 | Proteobacteria | Gammaproteobacteria | Enterobacteriales | Enterobacteriaceae | Escherichia/Shigella (0.94) |

L2FC, log twofold change in abundance; BM, mean abundance (base mean). Average confidence of the taxonomical assignment is indicated in parentheses.

**Supplementary Table S4.** Taxa identified as significantly differentially (Benjamin–Hochberg adjusted *p* < 0.05) abundant between ruminal fluid and ruminal tissue.

| BM | L2FC | L2FC SE | *P* | Phylum | Class | Order | Family | Genus |
| --- | --- | --- | --- | --- | --- | --- | --- | --- |
| Enriched in ruminal fluid | | |  |  |  |  |  |  |
| 11.25 | -22.37 | 6.14 | 2E-03 | Firmicutes | Bacilli | Bacillales | Bacillaceae_1 | Bacillus (0.87) |
| 25.65 | -24.47 | 6.14 | 7E-04 | Firmicutes | Bacilli | Bacillales | Bacillales_Incertae_Sedis_X | Thermicanus (1.0) |
| 386.88 | -25.75 | 2.98 | 1E-16 | Firmicutes | Bacilli | Lactobacillales | Streptococcaceae | Lactococcus (1.0) |
| 163.08 | -5.10 | 1.64 | 9E-03 | Firmicutes | Bacilli | Lactobacillales | Streptococcaceae | Streptococcus (1.0) |
| 100.66 | -6.92 | 2.57 | 0.03 | Proteobacteria | Alphaproteobacteria | Rhodobacterales (1.0) |  |  |
| 126.03 | -7.06 | 2.58 | 0.03 | Proteobacteria | Alphaproteobacteria | Rhodobacterales | Rhodobacteraceae | Paracoccus (0.9) |
| 7.02 | -21.01 | 6.13 | 4E-03 | Proteobacteria | Betaproteobacteria | Hydrogenophilales (1.0) |  |  |
| 5.63 | -22.33 | 6.13 | 2E-03 | Proteobacteria | Betaproteobacteria | Hydrogenophilales | Hydrogenophilaceae | Tepidiphilus (1.0) |
| 1267.42 | -3.35 | 0.94 | 3E-03 | Proteobacteria | Gammaproteobacteria | Enterobacteriales (1.0) |  |  |
| 839.66 | -3.33 | 0.90 | 2E-03 | Proteobacteria | Gammaproteobacteria | Enterobacteriales | Enterobacteriaceae | Escherichia/Shigella (0.94) |
| 28.24 | -22.10 | 4.65 | 3E-05 | Proteobacteria | Gammaproteobacteria | Enterobacteriales | Enterobacteriaceae | Obesumbacterium (0.42) |
| Enriched in ruminal tissue | | |  |  |  |  |  |  |
| 1243.30 | 1.02 | 0.42 | 0.01 | Actinobacteria (1.0) | |  |  |  |

L2FC, log twofold change in abundance; BM, mean abundance (base mean**)**. Average confidence of the taxonomical assignment is indicated in parentheses.

**Supplementary Table S5.** Taxa identified as significantly differentially (Benjamin–Hochberg adjusted *p* < 0.05) abundant between caecal fluid and caecal tissue.

| BM | L2FC | L2FC SE | *P* | Phylum | Class | Order | Family | Genus |
| --- | --- | --- | --- | --- | --- | --- | --- | --- |
| Enriched in caecal fluid | |  |  |  |  |  |  |  |
| 1394.36 | -1.35 | 0.51 | 0.02 | Firmicutes (1.0) |  |  |  |  |
| 1353.87 | -3.20 | 1.12 | 0.01 | Firmicutes | Bacilli | Lactobacillales (1.0) |  |  |
| 163.08 | -6.22 | 1.35 |  | Firmicutes | Bacilli | Lactobacillales | Streptococcaceae | Streptococcus (1.0) |
| 371.03 | -1.55 | 0.61 | 0.03 | Firmicutes | Clostridia | Clostridiales (1.0) |  |  |
| 1543.04 | -2.53 | 0.51 | 5E-06 | Proteobacteria (1.0) |  |  |  |  |
| 1267.42 | -2.30 | 0.79 | 0.0.1 | Proteobacteria | Gammaproteobacteria | Enterobacteriales (1.0) |  |  |
| 59.80 | -8.89 | 2.01 | 8E-05 | Proteobacteria | Gammaproteobacteria | Enterobacteriales | Enterobacteriaceae | Buttiauxella (0.48) |
| 839.66 | -2.81 | 0.75 | 1E-03 | Proteobacteria | Gammaproteobacteria | Enterobacteriales | Enterobacteriaceae | Escherichia/Shigella (0.94) |
| 28.24 | -27.55 | 3.86 | 4E-11 | Proteobacteria | Gammaproteobacteria | Enterobacteriales | Enterobacteriaceae | Obesumbacterium (0.43) |
| 454.15 | -3.88 | 0.73 | 2E-06 | Proteobacteria | Gammaproteobacteria | Pseudomonadales (1.0) |  |  |
| 436.92 | -4.38 | 0.74 | 7E-08 | Proteobacteria | Gammaproteobacteria | Pseudomonadales | Moraxellaceae | Acinetobacter (1.0) |
| Enriched in caecal tissue | |  |  |  |  |  |  |  |
| 498.21 | 2.39 | 0.58 | 4E-04 | Firmicutes | Bacilli | Bacillales (1.0) |  |  |
| 422.96 | 2.29 | 0.49 | 3E-05 | Firmicutes | Bacilli | Bacillales | Staphylococcaceae | Staphylococcus (1.0) |
| 7.02 | 18.30 | 5.11 | 2E-03 | Proteobacteria | Betaproteobacteria | Hydrogenophilales (1.0) |  |  |
| 5.63 | 18.32 | 5.11 | 2E-03 | Proteobacteria | Betaproteobacteria | Hydrogenophilales | Hydrogenophilaceae | Tepidiphilus (1.0) |
| 77.82 | 1.41 | 0.49 | 0.01 | Proteobacteria | Gammaproteobacteria | Xanthomonadales (1.0) |  |  |

L2FC, log twofold change in abundance; BM, mean abundance (base mean). Average confidence of the taxonomical assignment is indicated in parentheses.
